# Transcatheter mitral valve implantation with Tendyne System Ten Years since the First In-Human Implant A systematic review

**DOI:** 10.1186/s13019-023-02446-4

**Published:** 2023-11-10

**Authors:** Ahmed Ahmed, Tarek A. Abdel Aziz, Mohannad M. R. AlAsaad, Motaz Majthoob, Ahmed Toema

**Affiliations:** 1 Department of Cardiothoracic Surgery, Ain Shams University, Cairo, Egypt; 2 Dubai Hospital, Dubai, UAE; 3 Department of Cardiothoracic Surgery, Faculty of Medicine, Helwan University, Cairo, Egypt

**Keywords:** Mitral valve, Mitral regurgitation, Mitral calcification, Transcatheter mitral valve replacement, Tendyne valve

## Abstract

**Background:**

Transcatheter mitral valve replacement (TMVR) using the Tendyne™ valve is regarded as one of the most studied TMVR systems. The first human experience with the procedure was reported in 2013. The present study aims to systemically revise the published literature to document the global experience with TMVR using the Tendyne™ valve.

**Methods:**

The present review was conducted in line with the PRISMA statement on systematic reviews. Database included in the search process were Scopus, Web of Science and Pubmed. Search was processed using multiple keywords combinations and was adjusted to English literature only.

**Results:**

We included 26 articles in the final analysis reporting data from 319 patients. Patients recruited by the included studies comprised 192 males (60.2%) and 127 females (39.8%). In the studied patients, mitral annular calcification (MAC) was reported in 107 patients (33.5%). Preoperatively, MR grades 1,2 and 3–4 were reported in 3,5 and 307 patients respectively. Postoperatively, MR grades 1, 2 and 4 were reported in only 12, 3 and 1 patients respectively. Technical success was achieved in 309 patients (96.9%). Follow up durations widely varied among different studies from just days before discharge to 6 years. At the end of follow up, 79 patients died (24.8%) including 52 patients (16.3%) due to cardiovascular causes.

**Conclusions:**

Management of mitral valve disease using the Tendyne system appears to be a promising minimally invasive option for many high-risk patients with accepted procedural feasibility and safety profile.

## Introduction

Clinically significant mitral regurgitation (MR) is the most common valvular heart disease. The burden of the condition dramatically increases with older age [1]. Until recently, surgical repair and replacement were the standard therapeutic strategies. Considering the surgical high-risk profile of many patients, they were left without effective treatment options [2].

Over the past decade, technological advances continued to add multiple devices for transcatheter mitral valve repair and replacement with only few gained approvals by the healthcare authorities in Europe and the United States [3]. However, in view of the recent experience with these devices and the lack of long-term follow up studies, integrating their use into the standard treatment algorithms remains challenging [4].

Transcatheter mitral valve replacement (TMVR) using the Tendyne™ valve (Abbott Vascular, CA, USA) is regarded as one of the most studied TMVR systems. It’s a self-expanding prothesis with a double-frame design anatomically suited for the mitral annulus [5]. The first human experience with the procedure was reported in 2013 [6]. More recently, the Tendyne Global Feasibility Study has provided initial evidence of the procedural safety and efficacy over 2 years [7].

In Europe, the device has gained the CE mark [3]. In the United States, the ongoing randomized study “The Clinical Trial to Evaluate the Safety and Effectiveness of Using the Tendyne Mitral Valve System for the Treatment of Symptomatic Mitral Regurgitation (NCT03433274). is expected to test the advantage -if any- of Tendyne system over the standard conventional surgery [5].

### Preoperative planning and patient selection

Appropriate selection of patients suitable for Tendyne valve implantation requires multimodal cardiac imaging using transthoracic and transesophageal echocardiography and contrast-enhanced gated computed tomography. Imaging aims to evaluate the function and morphology of the mitral valve (MV) with special emphasis on severity of mitral annular calcification (MAC), angle of aortic mitral curtain and left ventricular outflow (LVOT) size [5, 8].

### The surgical technique

The Tendyne transcatheter valve is a self-expanding, fully retrievable and repositionable porcine valve. It’s composed of an inner circular stent frame and outer D-shaped stent frame. Both frames are made of nickel titanium alloy. The inner frame has only one size and has three leaflets. The outer frame has multiple sizes and has fabric cuff which sits at the annulus. The valve is anchored to the left ventricular apex by a tethered locking pad under guidance of fluoroscopy and transesophageal echocardiography under general anesthesia using the transapical approach through left minithoracotomy [8, 9].

The present study aims to systemically revise the published literature to document the global experience with TMVR using the Tendyne™ valve (Abbott Vascular, CA, USA).

## Methods

### Search methodology

The present review was conducted in line with the PRISMA statement on systematic reviews. Database included in the search process were Scopus, Web of Science and Pubmed. Search was processed using multiple combinations of the keywords (mitral valve, mitral regurgitation, transcatheter mitral valve, Tendyne system, Tendyne valve). Search was adjusted to include full-text journal articles published in English. Clinical studies of all types (Prospective, retrospective, comparative, etc.), case series and case reports were included. Retrieved records were published up to September, 2023. Selection criteria and search strategy and process were agreed by all co-authors.

### Inclusion criteria

All articles of all types published in English with at least essential preoperative characteristics and early postoperative course were included in the study.

### Exclusion criteria

Articles reporting Tendyne system data mixed with other TMVR systems or reporting data of patients included in other studies were excluded from final analysis.

### Data extraction and presentation

Data extracted from selected articles included type of article, country of origin, number and sex of patients, baseline Society of Thoracic Surgeons predicted risk of mortality (STS-PROM), left ventricular ejection fraction % (LVEF), presence of mitral annular calcification (MAC), degree of preoperative and postoperative mitral regurgitation at the last follow up, previous implants or procedures, technical success, other technical notes, duration of follow up, early (30-day) and later complications and cardiovascular and all-cause mortality. Outcome parameters were reported according to recommendations of the Mitral Valve Academic Research Consortium [10]. Obtained data were presented as number and percent, mean and standard deviation or median and interquartile range.

## Results

### Characteristics of the included studies

**Fig. 1:**
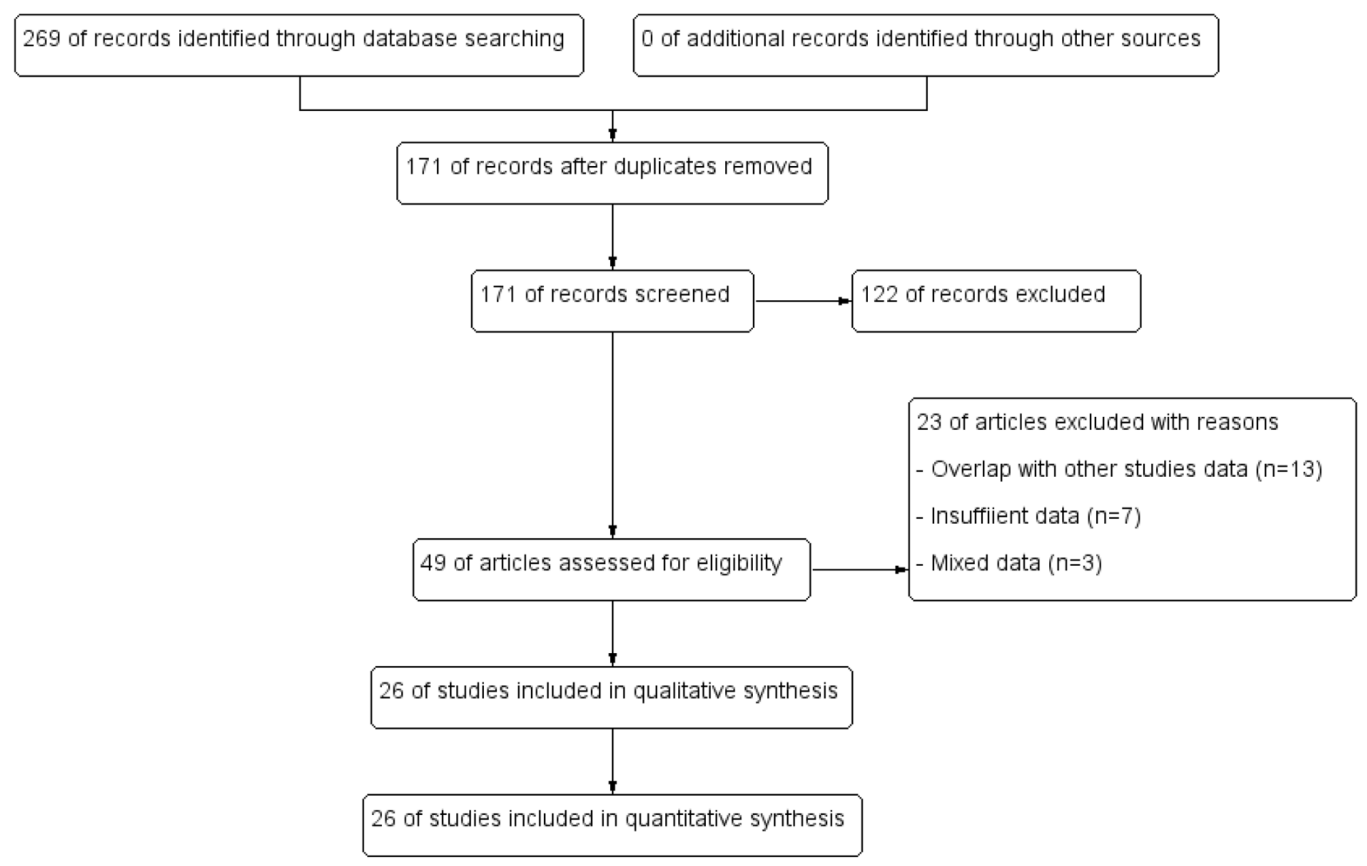
Study flow diagram

We could identify 269 records through search of the three databases using different keywords combinations. After thorough assessment of these records, we selected 49 articles for further assessment of full text if required. Finally, we included 26 articles in the final analysis reporting data from 319 patients (Fig. 1). Included studies types, country of origin and number of participants are listed in Table 1.

**Table 1:** Characteristics of included studies (N = 26)

	Country	Type	N
Aktuerk et al. [11]	Australia	Case report	1
Alarcon et al. [12]	Spain	Case report	1
Carnicer et al. [13]	Spain	Case report	2
Cerillo et al. [14]	Italy	Case report	1
Damian et al. [15]	Austria	Case report	1
Damian et al. [16]	Austria	Case report	1
Duncan et al. [17]	UK	Case series	5
Gossl et al. [18]	Multinational	Prospective	20
Grinberg et al. [19]	France	Case report	1
Hosadurg et al. [20]	USA	Case report	1
Muller et al. [7]	Multinational	Prospective	100
Norgren et al. [21]	Sweden	Case report	1
Nucera et al. [22]	Switzerland	Retrospective	24
Piperata et al. [23]	France	Case report	1
Polizzi et al. [24]	Italy	Case report	1
Pozzoli et al. [25]	Switzerland	Case report	1
Puehler et al. [26]	Germany	Case report	1
Ruge et al. [27]	Germany	Case report	1
Sorajja et al. [28]	USA	Case report	1
Sorajja et al. [29]	USA	Case report	1
Taramasso et al. [30]	Multinational	Retrospective	11
Ukaigwe et al. [31]	USA	Case report	1
Ukaigwe et al. [32]	USA	Case report	1
Wienemann et al. [33]	Germany	Comparative retrospective	15
Wild et al. [34]	Multinational	Retrospective/prospective	108
Wilde et al. [35]	Germany	Retrospective	17
**Total patients**			319

### Baseline characteristics of the studied patients

Patients recruited by the included studies comprised 192 males (60.2%) and 127 females (39.8%). Patients’ age, STS-PROM and LVEF % are shown in Table 2.

**Table 2:** Baseline clinical characteristics of the included patients (N = 319)

	N	Age (years)	Male/female n	STS-PROM %	LVEF %
Aktuerk et al. [11]	1	73	1/0	5.2	NA
Alarcon et al. [12]	1	76	1/0	NA	50.0
Carnicer et al. [13]	2	79,65	1/1	8.2, 28.2	42.0,37.0
Cerillo et al. [14]	1	86	0/1	NA	< 30.0
Damian et al. [15]	1	83	1/0	NA	51.0
Damian et al. [16]	1	80	1/0	NA	50.0
Duncan et al. [17]	5	64–87	3/2	15.4 ± 6.2	47.8 ± 10.2
Gossl et al. [18]	20	77.6 ± 5.9	9/11	8.1 ± 6.39	58.0 ± 9.0
Grinberg et al. [19]	1	85	0/1	6.0	35.0
Hosadurg et al. [20]	1	76	0/1	11.5	45.0–50.0
Muller et al. [7]	100	74.7 ± 8.0	69/31	7.8 ± 5.7	45.6 ± 9.4
Norgren et al. [21]	1	83	0/1	NA	60.0–65.0
Nucera et al. [22]	24	74.8 ± 7.8	16/8	7.7 ± 5.1	49.0 ± 12.5
Piperata et al. [23]	1	73	1/0	NA	40.0
Polizzi et al. [24]	1	77	1/0	NA	25.0
Pozzoli et al. [25]	1	82	1/0	NA	NA
Puehler et al. [26]	1	69	0/1	7.1	NA
Ruge et al. [27]	1	78	1/0	NA	NA
Sorajja et al. [28]	1	75	0/1	NA	NA
Sorajja et al. [29]	1	77	1/0	NA	NA
Taramasso et al. [30]	11	77.0 ± 6.0	4/7	9.0 ± 5.6	51.0 ± 9.0
Ukaigwe et al. [31]	1	69	0/1	NA	NA
Ukaigwe et al. [32]	1	78	1/0	NA	NA
Wienemann et al. [33]	15	80.3 (72.8–84.4)	10/5	NA	11 patients > 50.0
Wild et al. [34]	108	75.0 ± 7.0	62/46	7.2 ± 5.3	48.0 ± 12.0
Wilde et al. [35]	17	72.9 ± 9.4	8/9	NA	55.5 (52.2–58.0)
**Total**	319	-	192/127	-	-

LVEF: Left ventricular ejection fraction, STS-PROM: Society of Thoracic Surgeons predicted risk of mortality

### Mitral valve characteristics in the included patients

In the studied patients, mitral annular calcification (MAC) was reported in 107 patients (33.5%). Preoperatively, MR grades 1,2 and 3–4 were reported in 3,5 and 307 patients respectively. Postoperatively, MR grades 1, 2 and 4 were reported in only 12, 3 and 1 patients respectively (Table 3).

**Table 3:** Mitral valve characteristics in the included patients (N = 319)

	N	MAC	MR degree							
			Preoperative				Postoperative			
			1	2	3	4	1	2	3	4
Aktuerk et al. [11]	1	-	-	-	1	-	-	-	-	-
Alarcon et al. [12]	1	-	-	-	-	1	-	-	-	-
Carnicer et al. [13]	2	1	-	-	-	2	-	-	-	-
Cerillo et al. [14]	1	1	-	-	-	1	-	-	-	-
Damian et al. [15]	1	-	-	-	-	1	-	-	-	-
Damian et al. [16]	1	1	-	-	-	1	-	-	-	-
Duncan et al. [17]	5	-	-	-	-	5	-	-	-	-
Gossl et al. [18]	20	20	-	-	-	20	-	-	-	-
Grinberg et al. [19]	1	-	-	-	-	1	-	-	-	-
Hosadurg et al. [20]	1	1	-	-	-	1	1	-	-	-
Muller et al. [7]	100	-	-	-	100		4	-	-	-
Norgren et al. [21]	1	-	-	-	-	1	-	-	-	-
Nucera et al. [22]	24	9	1	-	8	15	2	1	-	-
Piperata et al. [23]	1	-	-	-	-	1	1	-	-	-
Polizzi et al. [24]	1	-	-	-	-	1	-	-	-	-
Pozzoli et al. [25]	1	-	-	-	-	1	-	-	-	-
Puehler et al. [26]	1	-	-	-	-	1	-	-	-	-
Ruge et al. [27]	1	1	-	-	-	1	-	-	-	-
Sorajja et al. [28]	1	1	-	-	-	1	-	-	-	-
Sorajja et al. [29]	1	-	-	-	-	1	-	-	-	-
Taramasso et al. [30]	11	1	-	-	11		-	-	-	-
Ukaigwe et al. [31]	1	1	-	-	-	1	-	-	-	-
Ukaigwe et al. [32]	1	-	-	-	-	1	-	-	-	-
Wienemann et al. [33]	15	14	1	-	-	14	1	-	-	1
Wild et al. [34]	108	47	1	5	34	64	3	1	-	-
Wilde et al. [35]	17	8	-	-	6	11	-	1	-	-
Total	319	107	3	5	307		12	3	-	1

MAC: Mitral annular calcification, MR: Mitral regurgitation

### Technical parameters in the included patients

Technical success was achieved in 309 patients (96.9%). Four patients were previously submitted to failed Mitraclip insertion. Other technical parameters are shown in Table 4.

**Table 4:** Technical parameters in the included patients (n = 319)

	N	Previous implants/procedures	Technical success	Technical notes
Aktuerk et al. [11]	1	-	1	-
Alarcon et al. [12]	1	-	1	LAMPOON procedure done
Carnicer et al. [13]	2	Failed Mitraclip*	2	-
Cerillo et al. [14]	1	-	1	-
Damian et al. [15]	1	-	1	Double aortic and mitral valves replacement
Damian et al. [16]	1	MV Annuloplasty	1	-
Duncan et al. [17]	5	-	5	-
Gossl et al. [18]	20	-	19	-
Grinberg et al. [19]	1	-	1	-
Hosadurg et al. [20]	1	-	1	-
Muller et al. [7]	100	-	97	-
Norgren et al. [21]	1	-	1	3 patients had concomitant ELASTA-Clip
Nucera et al. [22]	24		23	
Piperata et al. [23]	1	-	1	-
Polizzi et al. [24]	1	-	1	3D echocardiography used for monitoring
Pozzoli et al. [25]	1	Cadioband annuloplasty, Failed Mitraclip	1	First case of Valve-in-Ring implanting a Tendyne in Cardioband
Puehler et al. [26]	1	Aortic valve (twice)	1	-
Ruge et al. [27]	1	-	1	-
Sorajja et al. [28]	1	-	1	Pre-dilatation with balloon valvuloplasty
Sorajja et al. [29]	1	Failed Mitraclip	1	-
Taramasso et al. [30]	11	Aortic valve replacement (all patients)	11	Balloon valvuloplasty performed (n = 2)
Ukaigwe et al. [31]	1	-	1	Neo-left ventricular outflow tract modification with alcohol septal ablation
Ukaigwe et al. [32]	1	Failed Mitraclip	1	-
Wienemann et al. [33]	15	-	15	-
Wild et al. [34]	108	-	104	-
Wilde et al. [35]	17	-	16	-
Total	319	-	285	-

* Second ordered patient

ELASTA-Clip: electrosurgical laceration and stabilization of the clip, LAMPOON: Laceration of the Anterior Mitral leaflet to Prevent Outflow ObtructioN, HALT: Hypo-attenuated leaflet thickening

### Complications and mortality in the included patients

Follow up durations widely varied among different studies from just days before discharge to 6 years. At the end of follow up, 79 patients died (24.8%) including 52 patients (16.3%) due to cardiovascular causes. The most commonly reported complications included PVL, LOVTO and endocarditis (Table 5).

**Table 5:** Complications and mortality in the included patients (n = 319)

	N	Follow up	Complications		Mortality	
			Early	Late	Cardiovascular	All-cause
Aktuerk et al. [11]	1	18 months	PVL, hemolysis	-	-	1
Alarcon et al. [12]	1	Discharge	-	-	-	-
Carnicer et al. [13]	2	Discharge	-	-	-	-
Cerillo et al. [14]	1	16 months	-	PVL, hemolysis, Displacement, heart failure hospitalization	-	1
Damian et al. [15]	1	Discharge	-	-	-	-
Damian et al. [16]	1	60 days	-	-	-	-
Duncan et al. [17]	5	6 years	-	LVOTO (n = 1)	-	1
Gossl et al. [18]	20	1 year	-	PVL (n = 1), hemolysis (n = 1) heart failure hospitalization (n = 6) Stroke (n = 1)	4	8
Grinberg et al. [19]	1	8 weeks	PVL, LVOTO, Dislodgement, endocarditis	-	1	-
Hosadurg et al. [20]	1	1 year	HALT	-	-	-
Muller et al. [7]	100	2 years	-	Endocarditis (n = 5), PVL (n = 9), malposition (n = 4), thrombosis (n = 6), hemolysis (n = 3) Stroke (n = 5) PM (n = 8)	34	39
Norgren et al. [21]	1	3 months	LVOTO	-	-	-
Nucera et al. [22]		12 months	Hemolysis (n = 1) Stroke (n = 1) MI (n = 1)	MI (n = 1) Pacemaker (n = 1)	-	3
Piperata et al. [23]	1	8 months	-	Endocarditis	-	-
Polizzi et al. [24]	1	Discharge	-	-	-	-
Pozzoli et al. [25]	1	1 year	-	-	-	-
Puehler et al. [26]	1	Discharge	-	-	-	-
Ruge et al. [27]	1	Discharge	Ventricular tear	-	-	-
Sorajja et al. [28]	1	Discharge	-	-	-	-
Sorajja et al. [29]	1	Discharge	-	-	-	-
Taramasso et al. [30]	11	305.0 ± 270.0 days	-	PVL (n = 2)	-	3
Ukaigwe et al. [31]	1	Discharge	-	-	-	-
Ukaigwe et al. [32]	1	Discharge	Bleeding	-	-	-
Wienemann et al. [33]	15	320 days	PVL (n = 2)	PM (n = 4) Major/life threatening bleeding (n = 6) AKI (n = 3)	2	3
Wild et al. [34]	108	30 days	Device retrieval (n = 3), apical access site complications (n = 1)	PM (n = 2) Stroke (n = 3) AKI (n = 21) Major bleeding (n = 12) heart failure hospitalization (n = 14)	9	14
Wilde et al. [35]	17	370 (255–488)	-	Stroke (n = 1) Major bleeding (n = 1) Sepsis (n = 1)	2	6
Total	319	-	-	-	52	79

AKI: Acute kidney injury, HALT: Hypoattenuated leaflet thickening, LVOTO: Left ventricular outflow tract obstruction, MI, myocardial infarction, PM: Pacemaker, PVL: Paraventricular leak

## Discussion

Ten years after the first in-human implant of Tendyne system for management of MR, the present work sought to revise the published literature to evaluate how the technique evolved in terms of clinical value and technical development since its introduction. We included almost all the published articles whatever their types not to miss any piece of real-world experience with such a new devise.

As shown by our findings, the technology has been considered in more countries through Europe and North America. However, no reports from the rest of the world could be found to date. The barriers against wider use of such devices should be investigated. Probably, appropriate integration of minimally invasive devices in management of mitral valve disease into the standard treatment guidelines will encourage more surgeons to advocate their use.

In this review, we could easily recognize the promising potential of the Tendyne system in management of MR. After intervention was applied, only 1 patient was left with grade 4 MR while 10 and 2 patients had grade 1 and 2 MR respectively. These findings are limited by the short course of follow up. However, studies with the longest reported follow up duration including that of Duncan et al. [17], Gossl et al., [18], Muller et al., [7], Taramasso et al., [30], Wienemann et al., [33] and Wilde et al., [35] with a follow up duration ranging from almost one year up to six years showed also impressive results.

It’s clear that all patients included in this systematic review are of older age and most of them had deteriorated general condition and ventricular function and are unfit for surgery or other minimally invasive techniques. The availability of such minimally invasive procedure in this high-risk population adds a substantial value to treatment options. Notably, many patients included in our analysis were successfully submitted to Tendyne valve implantation after failed Mitraclip insertion [13, 25, 29, 32]. This highlights the value of the technology in complicated scenarios with limited options.

Remarkably, about one third of the patients evaluated in the present review had various degrees of MAC and almost all patients in the studies of Gossl et al. [18] and Wienemann et al. [33] had MAC. Apart from one patient, technical success was achieved and MR was resolved in all patients in both studies. After approximately 1 year of follow up, cardiovascular mortality was observed in 20.0% and 13.0% respectively.

Interestingly, the technology could also successfully in particularly challenging situations. Damian et al., [15] reported their experience with double aortic and mitral valves replacement. Also, Pozzoli et al., [25] elegantly documented their work with first case of valve-in-ring implanting a Tendyne in Cardioband.

Technically, the procedure showed remarkable procedural success rates and by time, surgeons could add many technical enhancements. In some situations, Laceration of the Anterior Mitral leaflet to Prevent Outflow ObtructioN (LAMPOON) procedure were done to improve the outflow tract [12]. In other cases, pre-dilatation with balloon valvuloplasty was found to be useful [30, 28] and neo-left ventricular outflow tract modification with alcohol septal ablation was also applied [31].

Paravalvular leak is considered the most common postoperative complications as noted by our review and in some instance may be so significant to induce systemic reactions [7, 18, 11]. In many cases, valve re-tensioning could successfully resolve the problem. Other reported complications included left ventricular outflow tract obstruction, hemolysis and endocarditis.

Generally, the Tendyne valve appears to have high rate of technical success and low rate of postoperative significant residual MR. However, similar to other TMVR devices, its use may be associated with relatively high rate of perioperative complications. In comparison with the transcatheter MV repair approaches e.g. MitraClip, the all-cause mortality and rehospitalization rate due to heart failure may be higher with TMVR devices [36].

In conclusion, management of mitral valve disease using the Tendyne system appears as a promising minimally invasive option for many high-risk patients with accepted procedural feasibility and safety profile. These conclusions may be limited by the short follow up period and lack of randomized controlled trials.

## Data Availability

The datasets used and analyzed during the current study are available from the corresponding author on reasonable request.

## Declarations

All methods were performed in accordance with relevant guidelines and regulations.
